# Shear-Dependent Agreement and Clinical Reclassification of Whole-Blood Viscosity Measurements: A Paired Comparison of Rheovis 2000A and Hemovister

**DOI:** 10.3390/diagnostics16081232

**Published:** 2026-04-20

**Authors:** Jongho Yi, Hong-Geun Jung, Seoung Joon Lee, Tae-Young Kim, Hahn Young Kim, Kyeong Ryong Lee, Hyun Suk Yang, Mina Hur

**Affiliations:** 1 Department of Laboratory Medicine, Konkuk University School of Medicine, Seoul 05030, Republic of Korea; keiel00@naver.com; 2 Department of Orthopedic Surgery, Konkuk University School of Medicine, Seoul 05030, Republic of Korea; jungfoot@hanmail.net (H.-G.J.); orthopediclee@gmail.com (S.J.L.); syty-chan@hanmail.net (T.-Y.K.); 3 Department of Neurology, Konkuk University Medical Center, Research Institute of Medical Science, Konkuk University School of Medicine, Seoul 05030, Republic of Korea; hykimmd@gmail.com; 4 Department of Emergency Medicine, Konkuk University School of Medicine, Konkuk University Medical Center, Seoul 05030, Republic of Korea; kuhemlkr@gmail.com; 5 Department of Cardiovascular Medicine, Konkuk University Medical Center, Research Institute of Medical Science, Konkuk University School of Medicine, Seoul 05030, Republic of Korea

**Keywords:** whole-blood viscosity, shear rate, hematocrit, scanning capillary viscometry, inter-device agreement, clinical reclassification

## Abstract

**Background/Objectives**: Whole-blood viscosity (WBV) is increasingly used in cardiovascular risk assessment; however, inter-device comparability may depend on shear-rate definition. We performed a paired comparison of two scanning capillary viscometers to evaluate shear-dependent analytical agreement and its impact on clinical classification. **Methods**: In 300 identical blood samples, WBV was measured using Rheovis 2000A and Hemovister. Systolic WBV was defined at 300 s^−1^ for both devices (shear-matched), whereas clinically defined diastolic WBV corresponded to 1 s^−1^ for Rheovis 2000A and 5 s^−1^ for Hemovister. Agreement was assessed using linear regression and Bland–Altman analysis. Hematocrit tertiles were examined as effect modifiers. Clinical agreement was evaluated using quadratic weighted Cohen’s κ. **Results**: Across matched shear rates (1000 to 1 s^−1^), Hemovister yielded consistently higher WBV values than Rheovis 2000A, with statistically significant inter-device differences at all shear levels except 1000 s^−1^. The magnitude of bias increased progressively as shear rate decreased, reaching −8.34 mPa·s at 1 s^−1^. Under shear-matched systolic conditions (300 s^−1^), the mean difference was −0.25 mPa·s (limits of agreement −1.72 to 1.22). In contrast, under clinically defined diastolic conditions (1 vs. 5 s^−1^), the mean difference was 14.54 mPa·s (3.93 to 25.15), increasing across hematocrit tertiles. Clinical agreement was fair for systolic (κ = 0.31; 95% CI 0.24 to 0.39) and moderate for diastolic WBV (κ = 0.44; 95% CI 0.37 to 0.51). Notably, among samples classified as high by Hemovister, 72.8% (systolic) and 54.0% (diastolic) were reclassified as normal by Rheovis 2000A. **Conclusions**: Inter-device agreement in WBV measurement is strongly shear-dependent. Although numerical divergence increases at low shear, categorical concordance may remain moderate when device-specific reference thresholds are applied. Harmonization of shear definitions and reference frameworks may therefore be essential for consistent cross-platform interpretation.

## 1. Introduction

Whole-blood viscosity (WBV) is a key hemorheological parameter reflecting the integrated effects of plasma viscosity, hematocrit, red blood cell deformability, and aggregation [1]. Elevated WBV has been associated with impaired microcirculatory perfusion and adverse cardiovascular outcomes, leading to growing interest in standardized, shear-dependent viscosity assessment [2,3,4,5].

Scanning capillary viscometry has emerged as a practical method for WBV measurement in clinical laboratories [6,7,8]. By estimating viscosity from pressure–flow relationships within a narrow capillary, these systems enable rapid characterization of shear-dependent rheological behavior across physiologically relevant flow conditions. WBV is typically reported at high shear rates (e.g., 300 s^−1^), representing systolic flow, and at low shear rates (e.g., 1–5 s^−1^), approximating diastolic or microcirculatory states [9,10]. Although based on a common physical principle, scanning capillary viscometers differ in automation level, capillary configuration, shear calibration, and data processing [11]. In particular, variation in the definition of low-shear conditions—such as reporting diastolic viscosity at 1 s^−1^ versus 5 s^−1^—may substantially influence measured values and their clinical interpretation under non-Newtonian conditions [9,10,12]. At present, no universally accepted low-shear standard exists for diagnostic WBV reporting, and platforms may adopt device-specific operational definitions based on internal calibration and historical validation datasets.

Hematocrit is a major determinant of WBV and may also modify inter-device differences [13,14,15]. At high shear rates, where blood behavior approaches near-Newtonian flow, methodological discrepancies may be attenuated. In contrast, at low shear rates, aggregation dynamics and cell–cell interactions are accentuated, potentially amplifying device-dependent bias in a hematocrit-dependent manner [14].

Previous inter-device comparisons have primarily emphasized correlation analysis. Early analytical evaluations comparing capillary-based systems demonstrated high correlation under controlled laboratory conditions, largely in plasma samples and matched measurement settings [16]. More recent comparisons across different viscometer types have confirmed that viscosity values may differ systematically according to measurement principles, even when applied to the same whole-blood sample [17]. However, such investigations have focused predominantly on analytical concordance and methodological differences, without evaluating shear-definition mismatch within scanning capillary platforms, hematocrit-dependent divergence, or the downstream impact on clinical categorization. Correlation alone does not ensure analytical agreement or clinical interchangeability, particularly when device-specific reference thresholds are applied.

Accordingly, we performed a paired comparison of two scanning capillary viscometry systems using identical blood samples to evaluate shear-dependent analytical agreement, assess the influence of hematocrit on inter-device differences, and quantify clinical reclassification based on device-specific reference thresholds.

## 2. Materials and Methods

### 2.1. Study Design and Analytical Framework

This cross-sectional, paired method-comparison study was conducted at the sample level to evaluate analytical agreement and clinical interchangeability between two scanning capillary viscometers. Between 27 March and 4 June 2025, consecutive blood samples submitted for routine WBV testing were included until 300 paired measurements were obtained. For each sampling episode, WBV was measured on both devices using the same blood specimen, thereby eliminating within-sample biological variability. The unit of analysis was the individual blood test. Although some individuals contributed more than one sample, each sampling episode was treated as representing an independent hematologic state. Demographic and laboratory data, including sex, age at sampling, and hematocrit, were retrieved from the corresponding blood draw.

### 2.2. WBV Measurements

WBV was measured using two scanning capillary viscometry systems: Rheovis 2000A (fully automated; Biorheologics Co., Ltd., Jeonju, Republic of Korea) and Hemovister (semi-automated; Ubiosis, Seongnam, Republic of Korea). Both devices estimate viscosity from pressure–flow relationships within a narrow capillary but differ in automation level, shear definition, and device-specific reference ranges. Systolic WBV was measured at 300 s^−1^ on both devices, representing high-shear flow conditions. Diastolic WBV was defined at 1 s^−1^ for Rheovis 2000A and at 5 s^−1^ for Hemovister. All viscosity values were expressed in mPa·s.

Quality control procedures were performed for Rheovis 2000A every 10 days using low-, mid-, and high-level control materials. Within-run and between-run precision data derived from these measurements are presented in Supplementary Table S1. For Hemovister, analytical performance characteristics were based on manufacturer specifications. Because both devices are based on physical measurement principles rather than chemical analytical processes, linearity was not evaluated. Carryover testing was not performed due to the use of disposable measurement kits.

All blood samples were collected in 3 mL VACUETTE K_3_ EDTA tubes (Greiner Bio-One, Kremsmünster, Austria) and processed according to standardized laboratory procedures. Samples were handled under controlled temperature conditions in a standardized laboratory setting and measured within 24 h after collection, in accordance with manufacturer-recommended protocols. All analyses were performed by trained medical technologists. The automated nature of the viscometers minimized operator-dependent variability.

### 2.3. Clinical Classification

WBV values were categorized as low, normal, or high according to device-specific and sex-specific reference ranges. For Rheovis 2000A, systolic (300 s^−1^) reference ranges were 3.66–5.41 mPa·s for males and 3.27–4.32 mPa·s for females; diastolic (1 s^−1^) reference ranges were 23.15–36.45 mPa·s for males and 18.20–27.36 mPa·s for females. For Hemovister, systolic (300 s^−1^) reference ranges were 3.5–4.1 mPa·s for males and 3.0–3.6 mPa·s for females; diastolic (5 s^−1^) reference ranges were 9.4–13.0 mPa·s for males and 7.6–11.1 mPa·s for females. Because the two systems define diastolic viscosity at different shear rates (1 s^−1^ vs. 5 s^−1^), clinical reclassification analyses reflect real-world device-specific interpretation rather than shear-matched analytical comparison.

### 2.4. Ethics Statement

This study was conducted in accordance with the Declaration of Helsinki and was approved by the Institutional Review Board of Konkuk University Medical Center (KUMC IRB 2025-06-019). The requirement for informed consent was waived because anonymized laboratory data obtained during routine clinical practice were analyzed.

### 2.5. Statistical Analysis

The target sample size of 300 paired measurements was determined pragmatically to ensure stable estimation of limits of agreement (LOA) and weighted κ statistics in accordance with a conventional method-comparison study design.

For clarity, comparisons were structured into two analytical frameworks: (1) shear-matched comparisons across identical shear rates (e.g., 1 vs. 1, 5 vs. 5, 300 vs. 300 s^−1^) to assess intrinsic analytical agreement, and (2) clinically defined comparisons based on device-specific low-shear definitions (1 vs. 5 s^−1^) to reflect real-world interpretative differences.

Continuous variables are presented as mean ± standard deviation (SD), and categorical variables as frequencies and percentages. Paired *t*-tests were used to compare WBV measurements between devices. Linear association was assessed using Pearson correlation coefficients. Analytical agreement was evaluated using Bland–Altman analysis, with mean bias defined as Rheovis 2000A − Hemovister and 95% LOA calculated as mean bias ± 1.96 × SD of the paired differences. To assess hematocrit-related modification, the inter-device difference (Rheovis 2000A − Hemovister) was analyzed as a continuous variable using Pearson correlation and simple linear regression. Bland–Altman analyses were additionally stratified by tertiles of hematocrit.

Clinical agreement in categorical WBV classification was assessed using weighted Cohen’s κ with quadratic weights and corresponding 95% confidence intervals (CI) [18]. Clinical reclassification was defined as a change in categorical interpretation when transitioning from Hemovister-based to Rheovis 2000A–based classification for the same sample.

All statistical tests were two-sided, and *p* < 0.05 was considered statistically significant. Statistical analyses were performed using IBM SPSS Statistics for Windows, version 28.0 (IBM Corp., Armonk, NY, USA) and MedCalc^®^ Statistical Software version 23.4.9 (MedCalc Software Ltd., Ostend, Belgium).

## 3. Results

A total of 300 paired samples were analyzed. The mean age at sampling was 61.0 ± 16.4 years, and 49.0% of samples were obtained from male individuals. The mean hematocrit was 38.9 ± 6.0%, with tertile cutoffs at 36.9% and 41.4%. Mean systolic and diastolic WBV values for each device are summarized in Table 1.

### 3.1. Shear-Dependent Analytical Agreement

**Figure 1:**
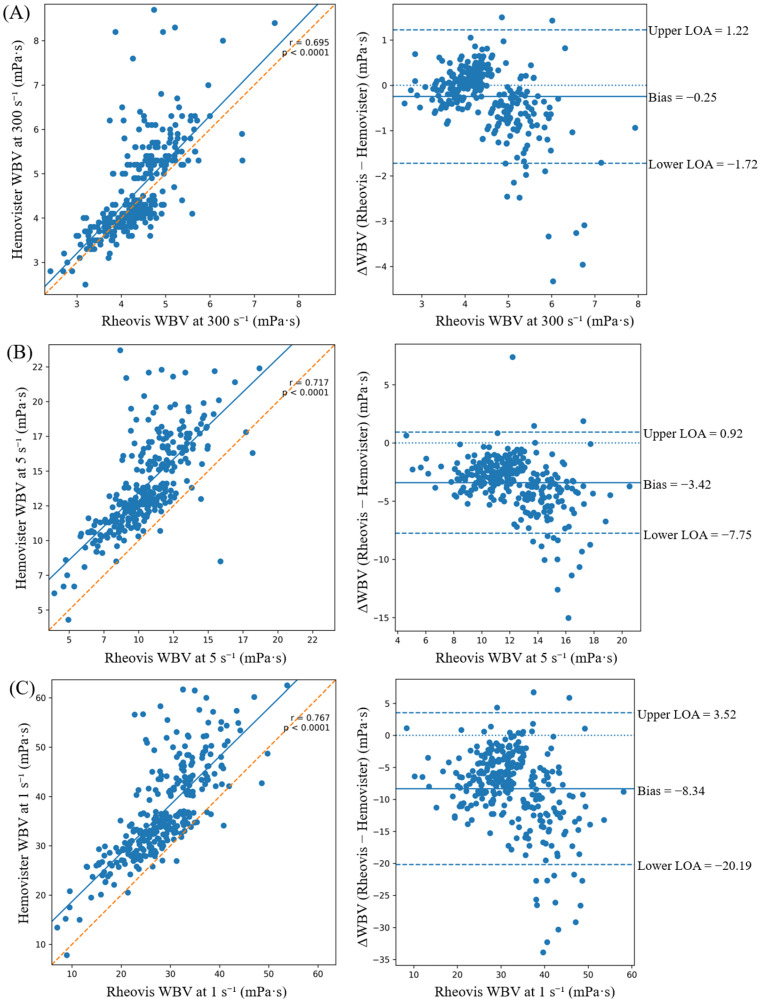
Correlation and Bland–Altman analyses comparing whole-blood viscosity (WBV) measured by Rheovis 2000A and Hemovister at (**A**) 300 s^−1^, (**B**) 5 s^−1^, and (**C**) 1 s^−1^. Scatter plots with linear regression (left panels) show Rheovis 2000A (*x*-axis) versus Hemovister (*y*-axis); the solid line represents the fitted regression line and the dashed line indicates the line of identity. Bland–Altman plots (right panels) depict the inter-device difference (Rheovis 2000A − Hemovister) plotted against Rheovis 2000A measurements. The solid line indicates the mean bias, and dashed lines represent the limits of agreement (LOA; mean bias ± 1.96 × SD).

Across the full shear spectrum (1000 to 1 s^−1^), Pearson correlation coefficients ranged from 0.65 to 0.77 (all *p* < 0.0001), indicating a strong positive association between devices (Table 1). Under shear-matched conditions, inter-device differences were small at high shear rates (0.06 mPa·s at 1000 s^−1^ and −0.25 mPa·s at 300 s^−1^). As the shear rate decreased, the magnitude of negative bias progressively increased, reflecting lower values measured by Rheovis 2000A compared with Hemovister. At 1 s^−1^ (shear-matched), the mean difference reached −8.34 mPa·s (95% CI −9.02 to −7.65; *p* < 0.0001). Bland–Altman analyses confirmed this shear-dependent divergence, with progressively wider dispersion at lower shear rates (Figure 1; Supplementary Table S2). Supplementary analyses (Table S2) further confirm that inter-device bias progressively increases with decreasing shear rate.

### 3.2. Clinically Defined Systolic and Diastolic WBV

**Figure 2:**
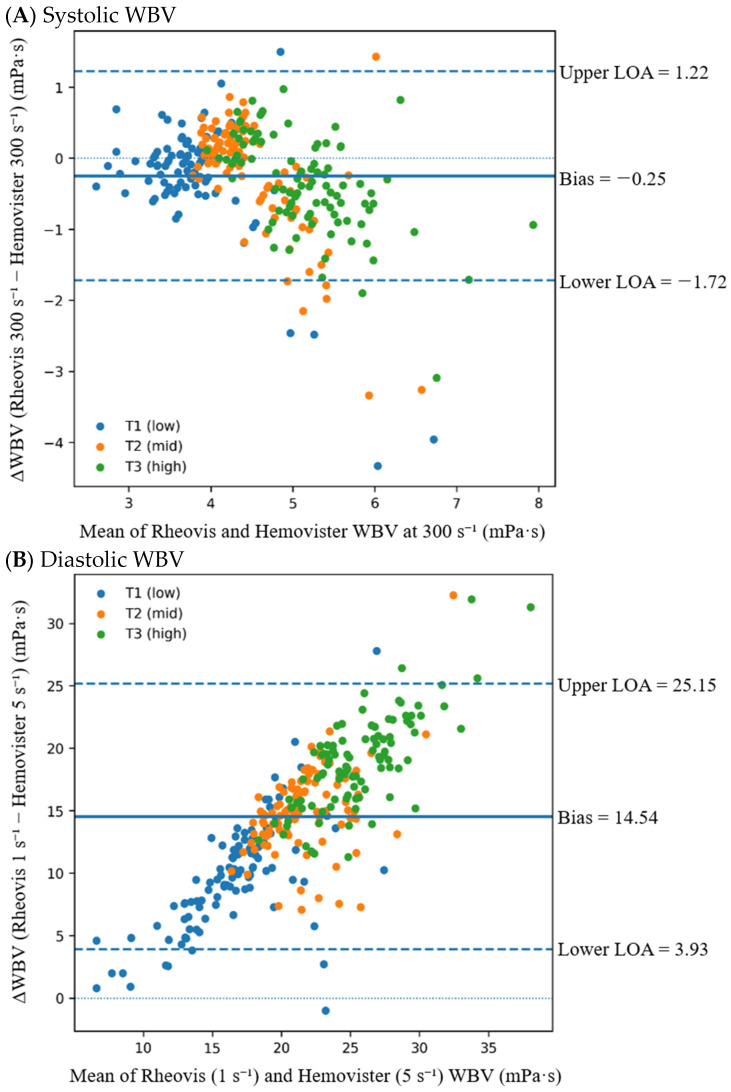
Bland–Altman plots for clinically defined systolic and diastolic whole-blood viscosity (WBV) stratified by hematocrit tertiles. (**A**) Systolic WBV was defined at 300 s^−1^ for both devices. (**B**) Clinically defined diastolic WBV was defined according to each device’s low-shear setting (Rheovis 2000A at 1 s^−1^ and Hemovister at 5 s^−1^). The *x*-axis represents the mean of paired measurements and the *y*-axis the inter-device difference (Rheovis 2000A − Hemovister, mPa·s). Hematocrit tertiles were defined using the 33rd and 66th percentiles of the study population (36.9% and 41.4%, respectively): T1 (≤36.8%), T2 (37.0–41.4%), and T3 (≥41.5%). The solid horizontal line denotes the overall mean bias; dashed lines indicate the limits of agreement (LOA), calculated as mean bias ± 1.96 × SD; the dotted line represents zero difference. Tertile-specific LOAs are provided in Supplementary Table S3.

In contrast to shear-matched analytical comparisons, clinically defined WBV comparisons reflect device-specific operational definitions rather than identical shear conditions. For systolic WBV, where both devices use a matched shear rate of 300 s^−1^, the mean bias was −0.25 mPa·s, with 95% LOA ranging from −1.72 to 1.22 mPa·s (Figure 2A).

However, when diastolic WBV was compared according to device-specific clinical definitions (Rheovis 2000A 1 s^−1^ vs. Hemovister 5 s^−1^), the mean bias was 14.54 mPa·s with markedly wider LOA (3.93 to 25.15 mPa·s) (Figure 2B), indicating substantial analytical divergence under low-shear definition mismatch.

### 3.3. Hematocrit-Dependent Modification

**Figure 3:**
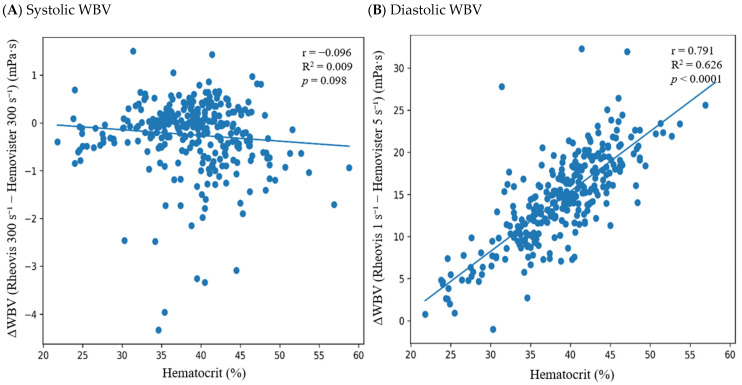
Association between hematocrit and inter-device difference in clinically defined systolic and diastolic whole-blood viscosity (WBV). (**A**) Relationship between hematocrit (%) and the inter-device difference (ΔWBV, defined as Rheovis 2000A − Hemovister) in systolic WBV at 300 s^−1^. (**B**) Relationship between hematocrit (%) and ΔWBV in clinically defined diastolic WBV (Rheovis 2000A at 1 s^−1^ and Hemovister at 5 s^−1^). Solid lines represent linear regression fits. No significant association is observed under shear-matched systolic conditions, whereas a strong positive relationship emerges under clinically defined low-shear conditions, indicating progressive amplification of inter-device differences with increasing hematocrit. This pattern supports the role of hematocrit as an effect modifier specifically under shear-mismatched low-shear conditions.

Under shear-matched conditions, hematocrit was not significantly correlated with inter-device differences at 300 s^−1^ (r = −0.096, *p* = 0.098; Figure 3A), 5 s^−1^ (r = −0.081, *p* = 0.161), or 1 s^−1^ (r = −0.045, *p* = 0.442). Stratified Bland–Altman analyses further demonstrated stable mean bias and LOA across hematocrit tertiles under shear-matched conditions (Figure 2A; Supplementary Table S3).

In contrast, under clinically defined diastolic conditions (1 s^−1^ vs. 5 s^−1^), inter-device difference in WBV (Rheovis 2000A − Hemovister) demonstrated a strong positive association with hematocrit (r = 0.791, *p* < 0.0001; Figure 3B). Each 1% increase in hematocrit was associated with a 0.712 mPa·s increase in inter-device difference (95% CI 0.650–0.775; *p* < 0.0001). Stratified Bland–Altman analysis further demonstrated progressive amplification of mean bias and widening dispersion across increasing hematocrit tertiles (Figure 2B; Supplementary Table S3).

Collectively, these findings indicate that hematocrit acts as an effect modifier under shear-mismatched low-shear conditions rather than as a general determinant of inter-device analytical bias.

### 3.4. Clinical Agreement and Reclassification Between Devices

For systolic WBV, overall categorical agreement between devices was fair (quadratic weighted κ = 0.31; 95% CI 0.24 to 0.39). Agreement was comparable between males and females, with overlapping confidence intervals (Table 2). Among samples classified as “high” by Hemovister, only 26.7% remained “high” under Rheovis interpretation, whereas 72.8% were reclassified as “normal” (Table 3). In contrast, all samples classified as “low” by Hemovister remained “low” under Rheovis interpretation (Table 3).

For diastolic WBV, overall concordance was 50.0%, corresponding to moderate agreement (quadratic weighted κ = 0.44; 95% CI 0.37–0.51). Agreement was similar in males (κ = 0.44; 95% CI 0.35–0.52) and females (κ = 0.42; 95% CI 0.30–0.54) (Table 2). Among Hemovister “high” samples, 54.0% were reclassified as “normal,” and 38.8% of “normal” samples were reclassified as “low” (Table 3). Overall, device-specific shear definitions and reference thresholds substantially altered categorical interpretation, even when measurements were derived from the same blood sample.

## 4. Discussion

In this paired, sample-level comparison of two scanning capillary viscometers, inter-device agreement proved to be definition-dependent rather than intrinsically device-dependent. When shear rates were analytically matched at high shear (300 s^−1^), numerical bias was minimal, and agreement was acceptable. In contrast, under clinically defined diastolic conditions—where Rheovis reports viscosity at 1 s^−1^ and Hemovister at 5 s^−1^—inter-device divergence was markedly amplified. This divergence increased progressively with higher hematocrit levels and translated into substantial categorical redistribution. These findings indicate that apparent analytical discordance at low shear primarily reflects definitional heterogeneity rather than measurement inaccuracy.

A key conceptual insight is that analytical divergence and clinical concordance are not interchangeable constructs. Importantly, the divergence observed under clinically defined conditions reflects differences in operational definitions rather than intrinsic measurement error, indicating that these comparisons represent interpretative rather than purely analytical discrepancies. Despite larger absolute bias under clinically defined diastolic conditions, quadratic weighted agreement remained in the fair-to-moderate range (κ = 0.31 for systolic and κ = 0.44 for diastolic WBV). This finding suggests that categorical stability can persist even when numerical values differ, provided that reference ranges are internally calibrated to device-specific shear definitions. Inter-device interchangeability should thus be evaluated within a reference framework rather than inferred solely from raw numerical bias.

Mechanistically, the amplification observed under low-shear conditions is consistent with the non-Newtonian behavior of blood [9,11,19]. As shear rate decreases, viscosity rises nonlinearly due to erythrocyte aggregation and intensified cell–cell interactions [1,11,14]. Under shear-matched conditions (300–300, 5–5, or 1–1 s^−1^), hematocrit was not independently associated with inter-device bias, supporting intrinsic analytical comparability. However, when diastolic WBV was compared using mismatched operational definitions (1 vs. 5 s^−1^), hematocrit acted as a potent effect modifier, magnifying divergence. These shear-dependent viscosity profiles are consistent with power-law behavior characteristic of non-Newtonian fluids, providing an additional rheological basis for interpreting the observed low-shear divergence as a manifestation of intrinsic flow properties rather than device-specific measurement error. Thus, hematocrit does not inherently generate device disagreement; rather, it accentuates definitional asymmetry under aggregation-dominant flow states. These findings suggest that direct mathematical conversion between different low-shear definitions may not be appropriate, as viscosity reflects complex nonlinear rheological behavior rather than a fixed proportional relationship.

Importantly, our findings do not establish the superiority of either 1 s^−1^ or 5 s^−1^ as a universal low-shear standard. Instead, they expose a broader conceptual issue: low-shear viscosity lacks cross-study harmonization. Prior investigations have variably defined diastolic WBV at 1 s^−1^—emphasizing extreme low-flow and microvascular aggregation phenomena [11,20]—or at 5 s^−1^, where more consistent associations with clinical outcomes have been reported [21,22]. Although some studies have reported viscosity at both 1 and 5 s^−1^ [9,12], cross-shear harmonization has rarely been the primary focus, and the selected shear rate is typically interpreted within study-specific analytical frameworks rather than evaluated for inter-platform definitional equivalence. Collectively, this pattern suggests that differences across studies reflect divergent physiological emphases rather than methodological hierarchy. Our data extend this observation by demonstrating that when devices operationalize diastolic viscosity using distinct shear definitions, the resulting asymmetry propagates through hematocrit-sensitive rheological amplification and manifests as clinically meaningful reclassification. In this context, low-shear viscosity should be understood as a definition-bound construct rather than a fixed physiological constant.

The clinical implications of this definitional dependency are nontrivial. A substantial proportion of samples categorized as “high” by Hemovister were reclassified as “normal” by Rheovis 2000A under both systolic (72.8%) and diastolic (54.0%) conditions. Although κ statistics indicated only fair-to-moderate agreement, this metric may underestimate systematic directional shifts and is influenced by category prevalence and marginal distributions. Even moderate κ values can mask clinically relevant redistribution when devices are used interchangeably without harmonized shear definitions and aligned reference ranges. This is reflected in our data, where higher concordance in females did not correspond to higher κ values, highlighting the influence of category distribution on κ interpretation. Consequently, device-specific interpretation may influence risk stratification, longitudinal monitoring, and cross-platform comparability in practice.

From an analytical perspective, both devices evaluated in this study are commercially available systems that have undergone prior analytical validation according to manufacturer specifications and established laboratory standards, including defined measurement ranges, precision characteristics, and controlled measurement conditions.

These findings highlight the need for greater standardization in low-shear WBV assessment. Given the substantial variability introduced by differing operational definitions, adoption of a unified low-shear reporting convention would improve comparability across studies and devices. Alternatively, reporting WBV at multiple low-shear rates (e.g., both 1 s^−1^ and 5 s^−1^) may provide a more comprehensive characterization of rheological behavior and enhance interpretability in both research and clinical contexts.

Several limitations warrant acknowledgment. First, from an analytical perspective, the present study was designed as a method-comparison analysis using clinical samples and did not include formal analytical performance validation according to Clinical and Laboratory Standards Institute (CLSI) guidelines (e.g., precision, linearity, and carryover) [23,24]. In addition, no independent reference method such as a rotational rheometer was included; therefore, the direction and magnitude of inter-device bias could not be anchored to an external analytical standard. Moreover, other rheological determinants—such as plasma protein composition and erythrocyte deformability—were not independently quantified [1,25,26]. Second, from a clinical perspective, reclassification analyses were not linked to prospective outcomes; therefore, the clinical consequences of redistribution remain uncertain. As a result, the clinical utility of reclassification could not be evaluated using outcome-based metrics such as net reclassification improvement or decision curve analysis. The observed divergence under diastolic conditions reflects real-world operational definitions and does not determine which low-shear setting carries greater physiological or prognostic validity. Third, from a study design perspective, this single-center study compared two specific scanning capillary viscometers, limiting generalizability to other platforms. In addition, the test-level analytical design and the limited representation of specific clinical subgroups precluded robust subgroup analyses across distinct disease conditions. Future prospective studies incorporating standardized low-shear definitions, external reference methods, and emerging analytical approaches [27,28], together with longitudinal clinical outcomes, will be essential to further clarify the analytical and prognostic relevance of WBV measurements across devices.

Despite these limitations, the paired design minimized biological variability and enabled direct assessment of inter-device agreement. By systematically contrasting shear-matched and clinically defined conditions, this study distinguishes intrinsic analytical comparability from definitional divergence. The combined use of Bland–Altman analysis, hematocrit-stratified modeling, and categorical reclassification provides a unified framework linking rheological behavior to clinical interpretation. Collectively, these findings suggest that harmonization efforts in hemorheology should prioritize explicit shear definitions and alignment of reference ranges, rather than assuming that identical numerical values are sufficient for clinical consistency. From a laboratory medicine perspective, these results highlight that analytical agreement and clinical interpretation must be considered together, particularly when operational definitions such as shear rate differ across platforms.

## 5. Conclusions

Shear-rate definition is a primary determinant of inter-device agreement in whole-blood viscosity measurement. While bias was negligible under shear-matched high-shear conditions (300 s^−1^), substantial divergence emerged under clinically defined low-shear settings (1 vs. 5 s^−1^) and was amplified by higher hematocrit. Importantly, numerical bias did not directly translate into categorical discordance, underscoring that analytical agreement and clinical concordance are distinct constructs. Harmonization of shear definitions and alignment of reference thresholds may therefore be essential for analytical standardization and consistent cross-platform interpretation.

## Figures and Tables

**Table 1:** Inter-device comparison of whole-blood viscosity across shear rates (*n* = 300).

Shear Rate (s^−1^)	Rheovis 2000A (Mean ± SD), mPa·s	Hemovister (Mean ± SD), mPa·s	Mean Difference, mPa·s (95% CI)	*p*-Value †	Pearson r	*p*-Value ‡
1000	4.26 ± 0.67	4.20 ± 1.07	0.06 (−0.03 to 0.15)	0.165	0.677	<0.0001
300	4.35 ± 0.70	4.59 ± 1.05	−0.25 (−0.33 to −0.16)	<0.0001	0.695	<0.0001
150	4.42 ± 0.74	4.98 ± 1.05	−0.55 (−0.64 to −0.47)	<0.0001	0.702	<0.0001
100	4.48 ± 0.77	5.28 ± 1.08	−0.80 (−0.89 to −0.71)	<0.0001	0.687	<0.0001
50	4.62 ± 0.83	6.00 ± 1.19	−1.38 (−1.48 to −1.28)	<0.0001	0.654	<0.0001
10	7.56 ± 1.55	10.15 ± 2.19	−2.59 (−2.76 to −2.42)	<0.0001	0.718	<0.0001
5	10.56 ± 2.35	13.98 ± 3.17	−3.42 (−3.67 to −3.16)	<0.0001	0.717	<0.0001
1	28.52 ± 7.36	36.85 ± 9.42	−8.34 (−9.02 to −7.65)	<0.0001	0.767	<0.0001

Data are presented as mean ± standard deviation (SD) (mPa·s). Mean inter-device differences (Rheovis 2000A − Hemovister) are shown with 95% confidence intervals (CI). † Derived from paired *t*-test. ‡ Derived from Pearson correlation analysis.

**Table 2:** Agreement of sex-specific clinical classification between Rheovis 2000A and Hemovister for systolic and diastolic whole-blood viscosity (WBV).

Phase	Sex	*n*	Concordance (%)	Quadratic Weighted κ (95% CI)
Systolic WBV	Overall	300	37.3	0.31 (0.24 to 0.39)
	Male	147	28.6	0.32 (0.24 to 0.40)
	Female	153	45.8	0.26 (0.13 to 0.38)
Diastolic WBV	Overall	300	50.0	0.44 (0.37 to 0.51)
	Male	147	40.1	0.44 (0.35 to 0.52)
	Female	153	59.5	0.42 (0.30 to 0.55)

Overall concordance (%) represents the proportion of samples classified into the same category by both devices. Agreement was assessed using quadratic weighted Cohen’s κ with 95% confidence intervals (CI). Agreement was interpreted as follows: κ < 0.20, poor; 0.21–0.40, fair; 0.41–0.60, moderate; 0.61–0.80, substantial; and >0.80, excellent [18]. Clinical categories (low, normal, and high) were defined according to device-specific, sex-specific reference limits. Systolic WBV was defined at 300 s^−1^ for both devices, whereas clinically defined diastolic WBV corresponded to 1 s^−1^ for Rheovis 2000A and 5 s^−1^ for Hemovister.

**Table 3:** Clinical reclassification of whole-blood viscosity (WBV) when switching from Hemovister to Rheovis 2000A.

(**A**) Systolic WBV				
**Hemovister (Rows) → Rheovis 2000A (Columns)**		**Low**	**Normal**	**High**
Overall (*n* = 300)	Low	9 (100.0%)	0 (0.0%)	0 (0.0%)
	Normal	17 (28.8%)	41 (69.5%)	1 (1.7%)
	High	1 (0.4%)	169 (72.8%)	62 (26.7%)
Male (*n* = 147)	Low	6 (100.0%)	0 (0.0%)	0 (0.0%)
	Normal	15 (40.5%)	22 (59.5%)	0 (0.0%)
	High	0 (0.0%)	90 (86.5%)	14 (13.5%)
Female (*n* = 153)	Low	3 (100.0%)	0 (0.0%)	0 (0.0%)
	Normal	2 (9.1%)	19 (86.4%)	1 (4.5%)
	High	1 (0.8%)	79 (61.7%)	48 (37.5%)
(**B**) Diastolic WBV				
**Hemovister (Rows)** **→** **Rheovis 2000A (Columns)**		**Low**	**Normal**	**High**
Overall (*n* = 300)	Low	9 (100.0%)	0 (0.0%)	0 (0.0%)
	Normal	26 (38.8%)	40 (59.7%)	1 (1.5%)
	High	2 (0.9%)	121 (54.0%)	101 (45.1%)
Male (*n* = 147)	Low	6 (100.0%)	0 (0.0%)	0 (0.0%)
	Normal	19 (51.4%)	18 (48.6%)	0 (0.0%)
	High	0 (0.0%)	69 (66.3%)	35 (33.7%)
Female (*n* = 153)	Low	3 (100.0%)	0 (0.0%)	0 (0.0%)
	Normal	7 (23.3%)	22 (73.3%)	1 (3.3%)
	High	2 (1.7%)	52 (43.3%)	66 (55.0%)

Values are presented as n (row-wise percentages that sum to 100% within each row). Rows indicate clinical categories according to Hemovister, and columns indicate categories according to Rheovis 2000A. Clinical categories (low, normal, and high) were defined according to device- and sex-specific reference limits. Systolic WBV was defined at 300 s^−1^ for both devices, whereas clinically defined diastolic WBV corresponded to 1 s^−1^ for Rheovis 2000A and 5 s^−1^ for Hemovister.

## Data Availability

The de-identified data supporting the findings of this study are not publicly available due to institutional and privacy restrictions but are available from the corresponding author upon reasonable request.

## Supplementary Materials

The following supporting information can be downloaded at: https://www.mdpi.com/article/10.3390/diagnostics16081232/s1, Table S1: Rheovis 2000A precision results; Table S2: Bland–Altman bias and limits of agreement for inter-device comparison of whole-blood viscosity across shear rates; Table S3: Bland–Altman agreement for clinically relevant whole-blood viscosity stratified by hematocrit tertiles.

## Abbreviations

The following abbreviations are used in this manuscript:

WBV	whole-blood viscosity
SD	standard deviation
CI	confidence interval
LOA	limits of agreement
κ	Cohen’s kappa
ΔWBV	inter-device difference in whole-blood viscosity
