# Supplementary material for: Shear-Dependent Agreement and Clinical Reclassification of Whole-Blood Viscosity Measurements: A Paired Comparison of Rheovis 2000A and Hemovister
Source: Diagnostics (Basel). 2026 Apr 20;16(8):1232. doi: 10.3390/diagnostics16081232 (PMC13114578; doi:10.3390/diagnostics16081232)
Supplement: Supplementary file 1 [file diagnostics-16-01232-s001.zip › Table_S1.pdf]

Supplementary Table S1. Rheovis 2000A precision results

Quality control material	Shear rate (s^{-1})	Coefficient of variation (%)		
		Within-run precision (%)	Between-run precision (%)	Total precision (%)
Low-level	Systolic viscosity (at shear rate 300 s^{-1})	3.71	2.90	3.53
	Diastolic viscosity (at shear rate 1 s^{-1})	4.40	2.55	4.04
Mid-level	Systolic viscosity (at shear rate 300 s^{-1})	3.29	4.45	3.60
	Diastolic viscosity (at shear rate 1 s^{-1})	3.42	3.70	3.49
High-level	Systolic viscosity (at shear rate 300 s^{-1})	4.06	3.86	4.01
	Diastolic viscosity (at shear rate 1 s^{-1})	3.43	4.26	3.65

Data are presented as coefficients of variation (%). Within-run and between-run precision were calculated from repeated quality control measurements. Total precision represents the combined variability from within-run and between-run measurements.
