# Supplementary material for: Shear-Dependent Agreement and Clinical Reclassification of Whole-Blood Viscosity Measurements: A Paired Comparison of Rheovis 2000A and Hemovister
Source: Diagnostics (Basel). 2026 Apr 20;16(8):1232. doi: 10.3390/diagnostics16081232 (PMC13114578; doi:10.3390/diagnostics16081232)
Supplement: Supplementary file 1 [file diagnostics-16-01232-s001.zip › Table_S2.pdf]

Supplementary Table S2. Bland–Altman bias and limits of agreement for inter-device comparison of whole-blood viscosity across shear rates

Shear rate (s^{-1})	*n*	Mean bias (Rheovis 2000A − Hemovister), mPa·s	Lower LOA (mPa·s)	Upper LOA (mPa·s)
1000	300	0.06	-1.48	1.61
300	300	-0.25	-1.72	1.22
150	300	-0.55	-2.03	0.92
100	300	-0.80	-2.35	0.74
50	300	-1.38	-3.15	0.39
10	300	-2.59	-5.58	0.40
5	300	-3.42	-7.75	0.92
1	300	-8.34	-20.19	3.52

Bias is defined as the mean inter-device difference (Rheovis 2000A − Hemovister). Limits of agreement (LOA) were calculated as mean bias ± 1.96 × SD of the paired differences. All values are expressed in mPa·s. Abbreviation: SD, standard deviation.
