# Supplementary material for: Shear-Dependent Agreement and Clinical Reclassification of Whole-Blood Viscosity Measurements: A Paired Comparison of Rheovis 2000A and Hemovister
Source: Diagnostics (Basel). 2026 Apr 20;16(8):1232. doi: 10.3390/diagnostics16081232 (PMC13114578; doi:10.3390/diagnostics16081232)
Supplement: Supplementary file 1 [file diagnostics-16-01232-s001.zip › Table_S3.pdf]

Table S3. Bland–Altman agreement for clinically relevant whole-blood viscosity stratified by hematocrit tertiles

Shear condition	Hematocrit tertile	Mean bias (mPa·s)	Lower LOA (mPa·s)	Upper LOA (mPa·s)
300 s^{-1} vs 300 s^{-1} (Systolic)	T1 (low)	-0.22	-1.78	1.34
	T2 (mid)	-0.19	-1.71	1.33
	T3 (high)	-0.35	-1.67	0.97
5 s^{-1} vs 5 s^{-1}	T1 (low)	-3.26	-8.37	1.85
	T2 (mid)	-3.45	-7.77	0.87
	T3 (high)	-3.54	-6.97	-0.10
1 s^{-1} vs 1 s^{-1}	T1 (low)	-8.32	-20.62	3.99
	T2 (mid)	-8.04	-20.40	4.32
	T3 (high)	-8.66	-19.55	2.23
1 s^{-1} vs 5 s^{-1} (Clinically defined diastolic)	T1 (low)	9.75	0.96	18.54
	T2 (mid)	15.05	8.34	21.76
	T3 (high)	18.90	11.47	26.34

Bias is defined as the inter-device difference (Rheovis 2000A − Hemovister), expressed in mPa·s. Limits of agreement (LOA) were calculated as mean bias ± 1.96 × SD of the paired differences. Systolic shear condition was defined at 300 s^{-1} for both devices. Clinically defined diastolic shear condition corresponded to 1 s^{-1} for Rheovis 2000A and 5 s^{-1} for Hemovister. Hematocrit tertiles were defined using the 33rd and 66th percentiles of the study population: T1 (≤36.8%, n = 100), T2 (37.0–41.4%, n = 102), and T3 (≥41.5%, n = 98). Abbreviation: SD, standard deviation.
